# Symbionts as Filters of Plant Colonization of Islands: Tests of Expected Patterns and Environmental Consequences in the Galapagos

**DOI:** 10.3390/plants9010074

**Published:** 2020-01-07

**Authors:** Jessica Duchicela, James D. Bever, Peggy A. Schultz

**Affiliations:** 1 Universidad de las Fuerzas Armadas-ESPE, Departamento de Ciencias de la Vida, Sangolquí 171103, Ecuador; 2 Indiana University, Biology Department, Bloomington, IN 47405, USA; 3 Department of Ecology and Evolution, and Kansas Biological Survey, Kansas University, Lawrence, KS 66047, USA; jbever@ku.edu; 4 Environmental Studies Program, and Kansas Biological Survey, University of Kansas, Lawrence, KS 66047, USA; pschultz@ku.edu

**Keywords:** arbuscular mycorrhizal fungi, habitat filtering, plant–soil feedback, soil aggregate stability, mycorrhizal response, native flora, introduced flora

## Abstract

The establishments of new organisms that arrive naturally or with anthropogenic assistance depend primarily on local conditions, including biotic interactions. We hypothesized that plants that rely on fungal symbionts are less likely to successfully colonize remote environments such as oceanic islands, and this can shape subsequent island ecology. We analyzed the mycorrhizal status of Santa Cruz Island, Galapagos flora compared with the mainland Ecuador flora of origin. We experimentally determined plant responsiveness and plant–soil feedback of the island flora and assessed mycorrhizal density and soil aggregate stability of island sites. We found that a greater proportion of the native island flora species belongs to families that typically do not associate with mycorrhizal fungi than expected based upon the mainland flora of origin and the naturalized flora of the island. Native plants benefited significantly less from soil fungi and had weaker negative soil feedbacks than introduced species. This is consistent with the observation that field sites dominated by native plant species had lower arbuscular mycorrhizal (AM) fungal density and lower soil aggregate stability than invaded field sites at the island. We found support for a mycorrhizal filter to the initial colonization of the Galapagos.

## 1. Introduction

Oceanic islands are distinguished by high endemism, low native diversity, and high vulnerability to invasion, but the drivers that sustain these patterns are not well understood. The theory of island biogeography has led to research on oceanic island species assembly determined by dispersal, establishment, and persistence mechanisms [1,2]. It is generally accepted that in order to colonize new islands successfully, new organisms need to adapt to the local conditions, including biotic interactions [3]. However, the understanding of the role of biotic interactions on island community assembly and their influence on oceanic islands ecosystem processes remains obscure, and its relevance has been highlighted as one of the 50 fundamental questions 50 years after the first appearance of the theory of island biogeography [4]. 

In this context, one of the most globally widespread and obligate symbionts of plant roots are arbuscular mycorrhizal fungi (AMF), phylum Glomeromycota. Mycorrhizal fungi arise from ancient symbioses between fungi and plant roots, which allow the fungi to receive plant-synthesized carbon while providing plants with increased nutrient and water foraging ability [5]. These individual plant growth effects can underlie community assembly and drive critical ecosystem processes. In particular, the symbiosis has a leading role in soil aggregate formation and long-term soil stabilization [6]. This association evolved very early in the evolution of terrestrial plants and likely played a pivotal role in supporting ancestral plant adaptation to the terrestrial environment [7,8]. Paleo-ecological studies suggest that historical range expansions did not rely solely on climatic shifts, highlighting that incompatibility with symbiont communities may have slowed host plant migrations in the Pacific Northwest [9]. Therefore, the structure of contemporary biota distribution might be explained, at least in part, by the host–symbiont interactions. 

Due to the long-distance dispersal limitation of arbuscular mycorrhizal fungi to the islands [10], an absence of mycorrhizal fungi on newly formed island land masses is likely. This would be expected to act as a filter as the establishment of plants that rely on these symbiotic interactions would be selected against, and therefore reduce the establishment of mycorrhizal dependent plants [11]. Although some plant species have evolved independence of AM fungi [12], all AM fungi are obligatory dependent on plant hosts. Therefore, mycorrhizal fungi have limited dispersal [13] and are unlikely to establish on newly formed islands devoid of host plants. As a result, plants that do not need mycorrhizal fungi are likely to be disproportionately represented in the initial colonists of islands. A mycorrhizal filter on initial plant colonization may decay over time as mycorrhizal fungi eventually arrive on islands [14] (p. 36), but the differential colonization rates can leave legacies that remain evident in modern flora and ecology of plant–symbiont interactions. This possibility was supported in an analysis of global floras, as native floras of oceanic islands were disproportionately represented by plant species that do not associate with mycorrhizal fungi compared to mainland floras [15]. Moreover, floras of naturalized species in oceanic islands were disproportionately represented by plant species that associate with mycorrhizal fungi, consistent with anthropogenic movement of plants with soils that has overcome the mycorrhizal filter [15]. This evidence suggests that mycorrhizal filter on initial plant colonization has left a legacy on the current ecology of the islands, but this possibility has not been tested.

The Galapagos archipelago is a unique system to test these relationships due to its oceanic origin, geographical isolation, and close relationship with adjacent continental flora [16]. Floristic and phylogenetic studies have suggested that most species of the Galápagos originated from South America [17,18,19]. However, the processes driving large-scale patterns of flora diversity on the islands, which are key considerations for attempts to conserve and restore terrestrial ecosystems, are still under discussion. 

Here, we provided the first empirical assessment of patterns of mycorrhizal association and their ecological consequences in Santa Cruz Island, Galapagos Archipelago to test the hypothesis that the lack of soil fungal symbionts, specifically AMF, acted as a filter in the initial colonization and subsequent ecology of the Galapagos Islands. We hypothesized that there would be evidence of a mycorrhizal filter in the Galapagos Islands if the proportion of native Galapagos floral species from plant families that do not associate with mycorrhizal fungi are significantly greater than the proportion in the native flora of continental Ecuador, where the Galapagos flora likely originated. As patterns of association with mycorrhizal floras may not reflect real differences in responsiveness to mycorrhizal fungi, we compared the responsiveness and plant–soil feedbacks of a suite of native and non-native plant species to the soil fungi in the Galapagos. Legacy effects of the original colonization filter would be demonstrated if the responsiveness of native plant species to mycorrhizal fungi were lower than plants arriving through anthropogenically-mediated invasion. Finally, we compared the density and diversity of AM fungi, as well as levels of soil aggregate stability, at sites dominated by native and non-native plant species, as evidence of legacy effects on patterns of ecosystem processes that might result from a mycorrhizal filter in the initial colonization of the Galapagos. 

## 2. Results

### 2.1. Flora Mycorrhizal Status Analyses

**Figure 1:**
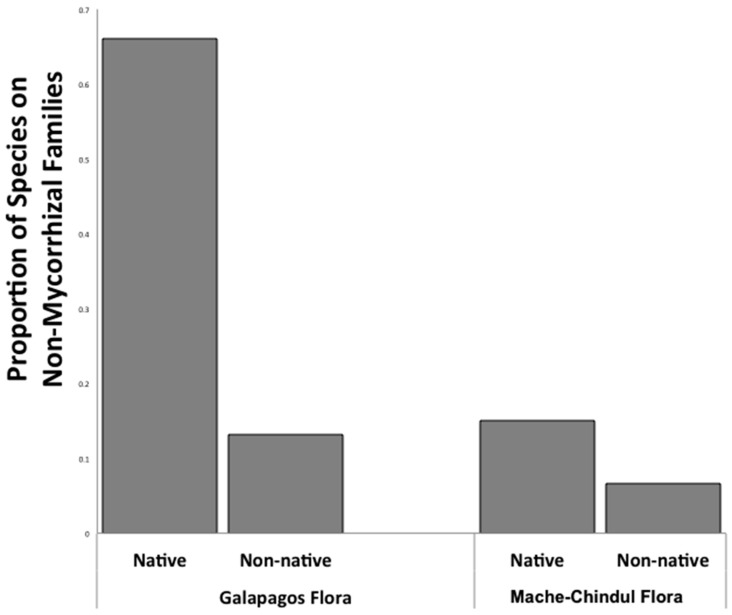
Pattern of nonmycorrhizal plant species in native and non-native flora of the highlands of Santa Cruz Island, Galapagos Archipelago, and flora of Mache-Chindul, mainland Ecuador.

The proportion of species from nonmycorrhizal families in the native flora of the Galapagos was significantly greater than that of the native flora on the mainland of Ecuador (chi sq = 61.94; *p* < 0.0001, Figure 1), consistent with the filtering of mycorrhizal dependent plant species from the initial colonization of the island. With the inclusion of non-native species in the floras, we found a significant interaction of mycorrhizal association by native by flora (chi sq = 5.11; *p* = 0.02). The analysis of 432 native and 145 introduced plant species in the flora of Galapagos demonstrated that a greater proportion of introduced species are members of plant families that commonly associate with mycorrhizal fungi as compared to native species (chi sq 32.19; *p* < 0.0001; Figure 1).

### 2.2. Plant Growth Response to Soil Fungi

**Figure 2:**
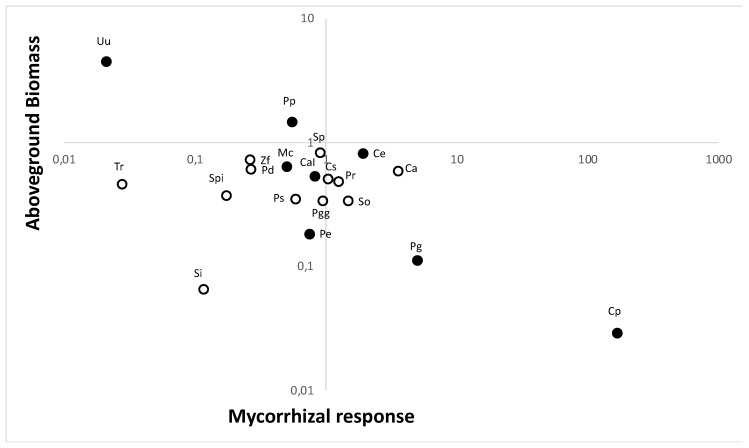
Scatterplot of log aboveground growth and log aboveground mycorrhizal response. Unfilled symbols represent native and filled symbols represent non-native plant species. Ca: *Chiococa alba*, CaI: *Coffea arabica*, Ce: *Cestrum auriculatum*, Cp: *Cinchona pubescens*, Cs: *Croton scouleri var. grandifolius*, Mc: *Momordica charantia*, Pd: *Pleuropetalum darwinii*, Pe: *Passiflora edulis*, Pg: *Psidium guajava*, Pgg: *Psidium galapageium*, Pp: *Pennisetum purpureum*, Pr: *Psychoria rufipes*, Ps: *Passiflora suberosa*, Si: *Sporobulus indicus*, So: *Senna occidentalis*, Sp: *Scalesia pedunculata*, Spi: *Senna pistaciifolia*, Tr: *Tournefortia rufo-sericea*, Uu: *Urtica urens*, Zf: *Zanthoxylum fagara.* Information about the flora botanical family, taxon origin, and growth habit is presented in Table S1.

Naturalized plant species responded more positively to soil fungi than native plant species (F1,17 = 4.9, *p* = 0.04). Woody plants responded more positively to soil fungi than non-woody plants (F1,17 = 4.6, *p* = 0.05). Naturalized plant species tended to be more heavily colonized than the native plant species (F1,14 = 6.4, *p* = 0.1). AMF root colonization did not significantly vary between woody and non-woody plants (F1,14 = 0.2, ns). Average plant size was a significant covariate of mycorrhizal responsiveness (F1,17 = 8.4, *p* = 0.01; Figure 2). Regression analyses indicate that fast-growing naturalized plant species were less responsive to mycorrhizal colonization than slower-growing naturalized plant species (F1,6 = 4.8, *p* = 0.07). Native plants were unresponsive regardless of the life form (F1,10 = 0.8, ns) and were less responsive to the live soil treatment than would be expected given their generally slow growth rates (F1,17 = 4.5, *p* = 0.05, Figure 2). Specific leaf area measurements were not significant. 

### 2.3. Plant–Soil Feedback Experiment

**Figure 3:**
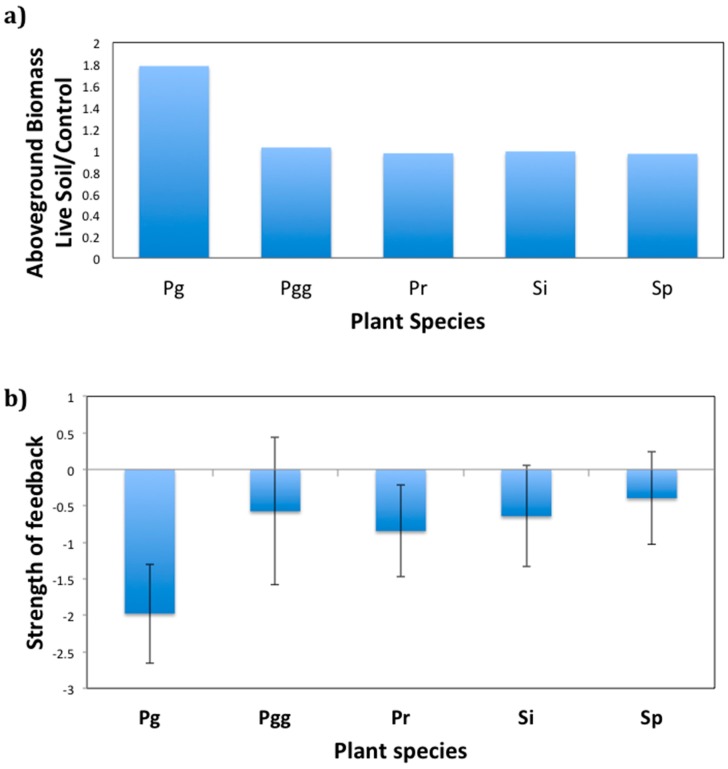
(**a**) Aboveground plant biomass and control ratios of feedback experiment and (**b**) variation in the strength of negative feedback through soil biota among the five seedling species using aboveground biomass. Errors and means that differ from zero are indicated by asterisks. Plant–soil feedback values give an indication of the effect of conditioned soil on plant growth, with positive values indicating better performance and negative values indicating low performance.

In evaluating the response of plant species to live versus sterile soils, we observed the same patterns as in the plant response experiment, where native species had lower response to live soil than introduced plant species (F1,182 = 5.26, *p* = 0.02, Figure 3a). We also found that the introduced plant species had stronger negative feedbacks than the four native plant species (F1,182 = 6.01, *p* = 0.01). The four native plant species did not grow significantly differently in soil communities trained by conspecifics compared to soil communities trained by other plant species, whereas the non-native plant species were smaller when grown with conspecific soil communities compared to its growth in soil from other plant species (F1,182 = 8.43, *p* = 0.004, Figure 3b) (Analysis of Variance and means of Feedback Test experiment are shown in Tables S2 and S3 of Supporting information).

### 2.4. Field Patterns

**Figure 4:**
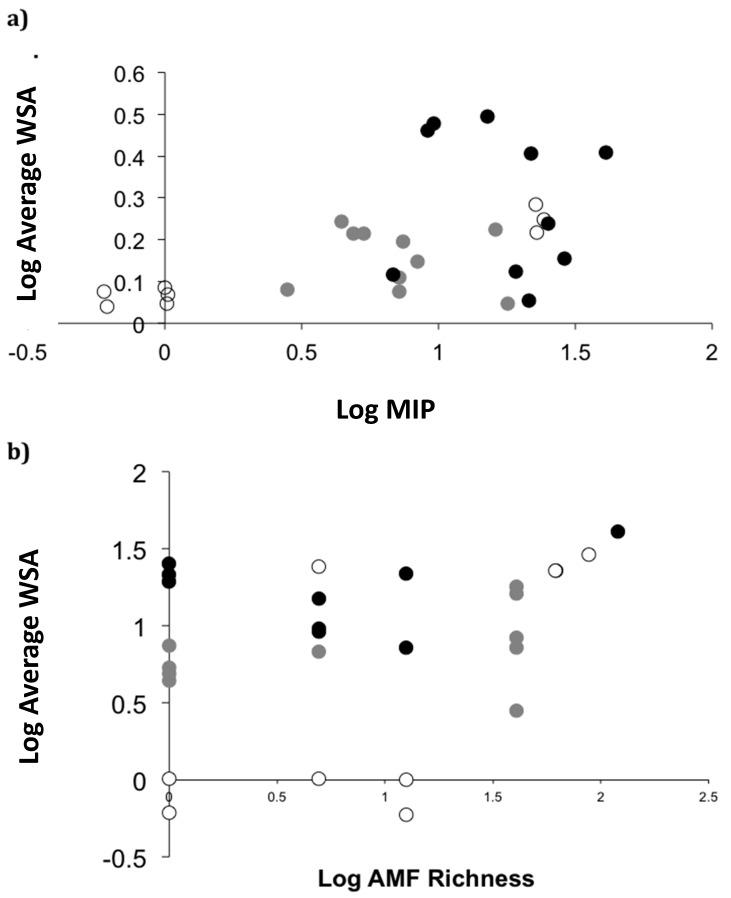
Scatterplot of (**a**) log of Mycorrhizal Inoculum Potential (MIP) and log of average Water Stable Aggregates (WSA) and (**b**) log arbuscular mycorrhizal richness and log average WSA. Unfilled dots represent sites with native vegetation, gray dots represent sites with agricultural disturbance, and filled black dots represent plant invaded sites.

When analyzing the soil properties and mycorrhizal inoculum potential of sites dominated by native and non-native plants, we found that the sites dominated by non-native plant species had a greater proportion of soil stable aggregates (WSA) than sites dominated by native species (F2,22 = 14.1, *p* < 0.0001). Sites with non-native plant species also tended to have higher AM fungal inoculum potential (MIP, F2,22 = 7.3, *p* = 0.08). There were significantly higher WSA and MIP in soils with non-native plants than native plants, whereas AM fungal species diversity did not vary consistently across land-use histories (F2,22 = 0.2, ns). Soil samples averaged two AM fungal species across all soil samples. The proportion of WSA was positively correlated with MIP and AM fungal richness (Figure 4). The model that best predicted WSA (AICC = 2.6, next lowest AICC = 7.4) was soil nitrate concentration (F1,25 = 72.3, *p* < 0.0001) and MIP (F1,25 = 7.8, *p* < 0.01), with WSA decreasing with soil nitrate concentration and increasing with MIP.

## 3. Discussion

Although some plant species have evolved autonomy from AM fungi [12], all AM fungi are obligately dependent on plant hosts, setting up expectations for the initial colonization of oceanic islands by plants with low dependence on AM fungi. We found support for the hypothesis that dependence on mycorrhizal fungi acted as a filter during the colonization of the Galapagos Islands. This support comes from analyses of floras, where a much greater proportion of plant species native to the Galapagos Islands are from plant families that do not associate with mycorrhizal fungi compared to the floras from areas of mainland Ecuador, which are the likely source of the Galapagos flora [18,20,21]. This result is consistent with analyses of global flora patterns [15] and expands upon the findings of Carvajal-Endara et al. (2017) [21] that colonizing species of the Galapagos have specific niche requirements that include biotic dimensions of plant niche as a habitat filter. Our results are consistent with the initial colonization of the islands favoring plants that are capable of succeeding in the absence of AM fungi. This would be expected given the limited ability of AM fungi to disperse long distances and the large distance (~1000 km) separating the Galapagos from the South American continent. The low total AM fungal richness observed in our field samples was less than half of comparable sampling areas on mainland [22]. This finding is consistent with expectations of limited dispersal AM fungi and the young age of the islands, which are some of the most newly formed volcanic islands on earth at approximately one million years old. The sampling of the Hawaiian Islands showed evidence of mycotrophy in native plant species [23,24]. However, this includes old islands up to approximately 65 million years old. The older islands might have had time to accumulate mycorrhizal plants, explaining why the results of Koske et al. (1992) [23] are not consistent with our results. The colonization by AM fungi and subsequent colonization by AM fungal-dependent plants were more likely over that time span. An alternative explanation to the colonization order hypothesis could be that rock of the newly formed Galapagos Islands was high in phosphorus and therefore AM fungi were not necessary. Patterns of anthropogenic invasion do not support this alternative hypothesis, however, as anthropogenically introduced plant species associated with and respond to AM fungi. 

We found that plants introduced since the onset of human colonization of the Galapagos Islands are more likely to depend on AM fungi than native plant species. Anthropogenically introduced plant species are more likely to be from plant families that associate with AM fungi than native plant species to the Galapagos. Moreover, controlling for plant family, anthropogenically introduced plant species are more responsive to inoculation with soil fungi (compared to fungicide treatment or sterilized soil inoculum) than native species to the Galapagos. Although plant response to live soil fungi could be due to soil pathogens, we did not find support for soil pathogens contributing to the difference in response of native and anthropogenically introduced plant species, as we did not observe significant negative plant soil feedbacks in native plant species. Rather, we observed stronger negative plant–soil feedbacks in the introduced plant species, *Psidium guajava*, than in four native plant species (Figure 3b). Interestingly, this pattern is the opposite of the one observed on mainland studies, where native species have stronger negative feedbacks [25]. Together, our results suggest that the limitation of mycorrhizal fungi on the Galapagos Islands could have been overcome following human settlement, perhaps through direct introduction of soil fungi via transplanted crops with live soil. Novel plant pathogens could also have been brought over by transplanted crops, contributing to our observations of negative plant–soil feedback in naturalized plant species.

**Figure 5:**
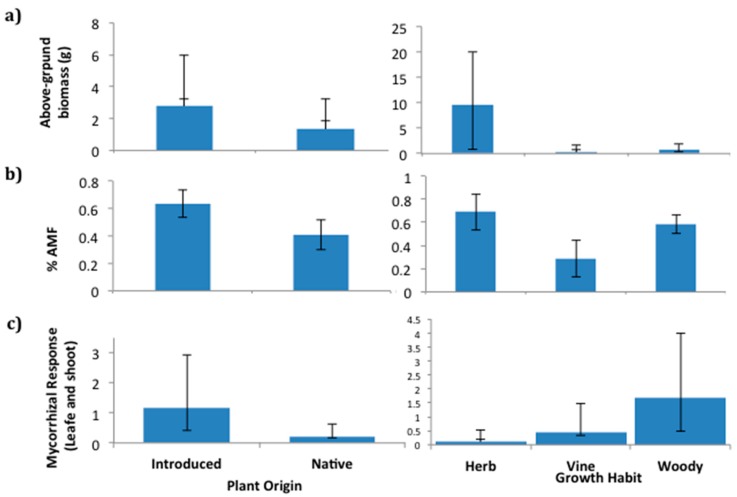
The mean and standard error of the (**a**) aboveground (leaves and shoots) biomass, (**b**) rate of root arbuscular mycorrhiza colonization, and (**c**) mycorrhizal response of plant species by plant origin: Introduced and native (left panels) and growth habit: Herb, vine, and woody (right panels). The aboveground mycorrhizal response was calculated as the ratio of the leaf and shoot biomass gained in live soil over the same biomass gained in soil treated with fungicide.

In anthropogenically introduced plant species in the Galapagos, fast-growing plant species have low responsiveness to mycorrhizal fungi, while slow-growing plant species respond positively. A similar pattern was observed on mainland sites in native plants of North America [26,27], as would be expected given that both low dependence on AM fungi and rapid growth rates would aid in colonization of disturbed lands. However, native plants to the Galapagos do not show this relationship. Rather they have low responsiveness to mycorrhizal fungi, even though they consistently grow slowly (Figure 5a). The absence of this relationship in native plants suggests that mycorrhizal fungi may play a different role in plant succession on the island than what has been found in mainland plants. On mainland, changes in density and composition of mycorrhizal fungi can be expected to differentially influence the competitive ability of late successional plant species, thereby contributing to succession [26,28]. However, mycorrhizal fungi may not play that role in the Galapagos Islands. For both native and non-native species, woody plants were more responsive to AM fungi than non-woody plants (Figure 2), which is consistent with the results of a prior meta-analysis [29]. 

In correlation with their greater responsiveness to AM fungi, roots of non-native plants on the Galapagos Islands tended to have greater colonization by AM fungi than comparable native plant species. This greater level of mycorrhizal colonization suggests that non-natives are better hosts for AM fungi than native plant species. As in previous observations of low levels of mycorrhizal colonization in roots of native plants from the island [30], we also observed that sites dominated by non-native plant species tended to have greater AM fungal densities than sites dominated by native plant species. The greater densities of AM fungi in invaded sites may fuel sustained future success of non-natives in these areas, as non-natives benefit more from AM fungi than natives. Such positive feedback through AM fungal composition would have important implications for the management and restoration of the native Galapagos flora. Evidence of positive feedback through changes in AM fungal density has been reported in mainland North America, but with an opposite orientation. Analyses of flora in California showed that non-native species are more likely to be from non-mycorrhizal families than native species [31] and this is consistent with analyses of global floral patterns [15]. Experimental tests confirmed that dominance by non-natives decreases the densities of mycorrhizal fungi, which then inhibit establishment of native plant species [32]. A similar dynamic has been demonstrated with non-native garlic mustard in woodlands of North America [33], which, as a nonmycorrhizal species, can provide a competitive advantage to the invader over native mycorrhizal plants. Recent studies have been ambiguous as to whether non-native plant species are expected to consistently differ in their dependence on mycorrhizal fungi compared to native plant species [31,34]. Our results identified that expectations for the relative responsiveness of native and non-native plant species, and the ecological consequences of these differences, may depend upon biogeographic history of the area under investigation. 

Non-native plants can have strong impacts on soil microbial communities and soil function. In the Galapagos, we found that areas dominated by non-natives have higher aggregate stability than areas dominated by native species. High aggregate stability was correlated with the measures of AM fungal inoculum potential and AM fungal species richness. A similar dependence of soil aggregate stability on mycorrhizal inoculum potential and fungal composition has been found in studies in North and South America [6,35]. However, in these mainland studies, native dominated communities had higher inoculum potential and higher soil aggregate stability consistent with the high dependence of native plant species in these grasslands [26,32,36].

The contrasting dynamics of plants and AM fungi between the mainland and the Galapagos Islands presents a dilemma with regard to restoration and ecosystem management. On the mainland, restoration of native plants can be enhanced by inoculation with mycorrhizal fungi, particularly native mycorrhizal fungi [26,37,38], and we expect that ecosystem function as represented by aggregate stability would also be enhanced [6]. However, the restoration of native plant species and enhancement of ecosystem functions may not go hand in hand in the Galapagos. Our results suggest that inoculation with AM fungi would decrease the dominance of native plants while enhancing aggregate stability. Alternatively, inhibition of AM fungal density in areas dominated by non-natives may facilitate the transition to native but decrease aggregate stability. 

We found consistent support for contemporary legacies of a mycorrhizal filter on initial plant community assembly of the Galapagos Islands from analysis of floras, comparative analysis of contemporary plant–microbial ecology, and field surveys of plants, mycorrhizal fungi, and soil properties. Although this work provides promising support for a novel set of hypotheses on island biogeography, more needs to be done to confirm these patterns and processes. For example, our comparative analyses of floras were not inclusive of all potential continental sources for plant species colonization of the Galapagos [21]. Moreover, the Galapagos flora has likely been influenced by multiple processes that we did not consider, including volcanic activity, changes in sea levels, and island subsidence [39,40,41], each of which could potentially interact with patterns of mycorrhizal associations of plant species. We explored island ontogeny, which might capture some of this complex history. Although this factor was additionally significant, it did not change our conclusions regarding the mycorrhizal filter on plant establishment. Reconstruction of the colonization history of the mycorrhizal fungi of the Galapagos Islands through genetic and phylogenetic approaches could provide an independent test of the mycorrhizal filter hypothesis. Our results provide motivation for further work testing the extent of patterns consistent with mycorrhizal filters and their ecological legacies that are evident on other oceanic islands. 

In summary, our results provide support for the hypothesis that dependence on plant symbionts can filter the initial plant establishment on islands. Analyses of contemporary native Galapagos flora are consistent with the plant species initially colonizing the Galapagos Islands being less likely to associate with arbuscular mycorrhizal fungi compared to their likely flora of origin. Moreover, we found evidence that the legacy of this initial filter on colonization structures the modern ecology of the islands. In particular, we found that the plant species established following human colonization of the islands have a greater association with, and greater responsiveness to, arbuscular mycorrhizal fungi than the native plant species. Areas dominated by invasive plant species have greater densities of AM fungi and greater soil aggregate stability than areas dominated by native plant species, providing evidence that plant-AMF colonization can transform ecosystem properties generating persistent legacies that need to take in account for ecosystem restoration. 

Our results are also consistent with mycorrhizal fungi being introduced to the island with human colonization and then facilitating invasion and ecosystem transformation by non-native plants. Further research is needed to substantiate this co-invasion hypothesis, as well as the operation of the mycorrhizal filter in initial plant colonization and the ecosystem legacy.

This study represents the first attempt to analyze the mycorrhizal status and experimentally test mycorrhizal responsiveness in the Galapagos flora. Our results revealed that incorporating both plant status and mycorrhizal responsiveness data into future analyses that could provide a more detailed insight into the influence of mycorrhiza on Galapagos flora and contribute a basis for understanding the role of mycorrhizas symbiosis in ecosystems functioning the face of global change. This is particularly relevant when assessing plant invasion, which is still commonly studied at the individual plant species level. Yet, interactions with symbiotic fungi affect plant survival and establishment and potentially contribute to invasion success. The consistency of our analysis of the Galapagos flora with the results from an analysis of a global database [15], suggests that the patterns found here for island versus continental flora mycorrhizal status are general. The balance of escaping from enemies and finding a suitable mycorrhizal partner may influence the ecology of islands in other parts of the globe.

## 4. Materials and Methods 

### 4.1. Study System

**Figure 6:**
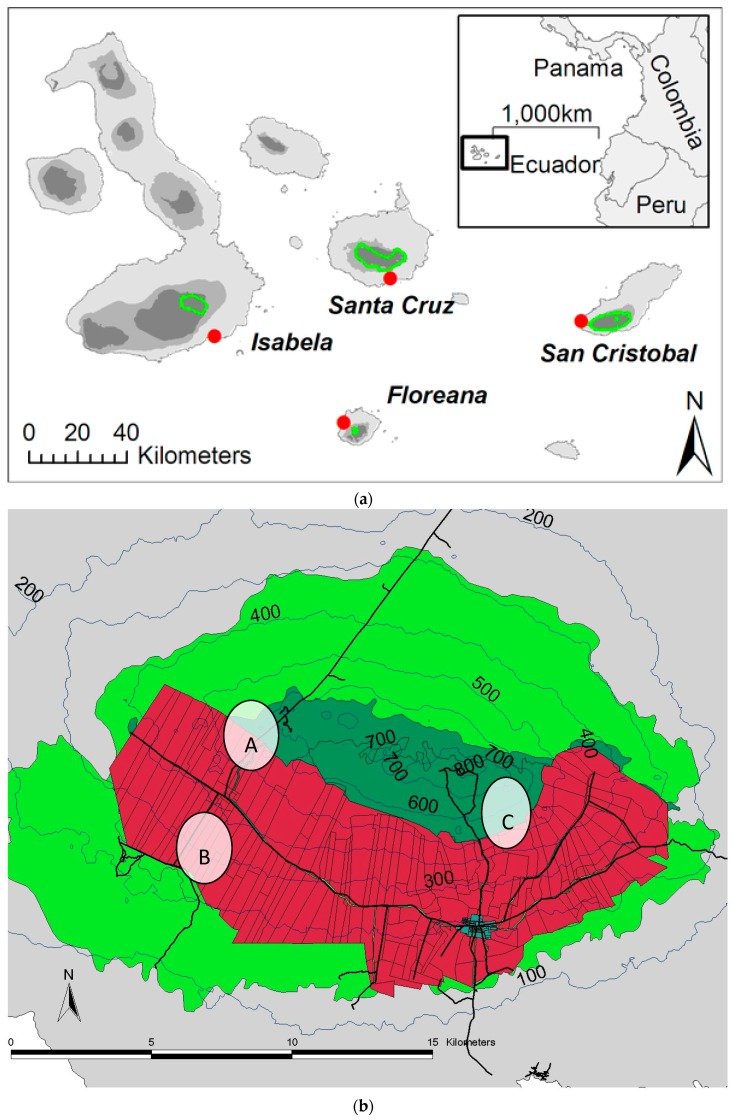
(**a**) Geographical location of Galápagos in relation to mainland Ecuador. Figure 6. Location map of Galapagos, showing the broad climatic zones (light: arid, medium: transition, dark: humid) from [48] Guézou A, Trueman M, Buddenhagen CE, Chamorro S, Guerrero AM, et al. (2010) An Extensive Alien Plant Inventory from the Inhabited Areas of Galapagos. PLOS ONE 5(4): e10276. https://journals.plos.org/plosone/article/figure?id=10.1371/journal.pone.0010276.g001. Copyright @2010Guézou et al. (**b**) Highlands of Santa Cruz Island shows the current agricultural zone (in red) and the dots indicate the location of the sites in study: (A) Granillo Alto and Granillo Bajo, (B) Fundar Galapagos, and (C) Cerro Mesa4.2. Flora Mycorrhizal Status Determination.

The study was conducted on the Galapagos Islands, an oceanic archipelago of volcanic origin located 1000 km west of mainland Ecuador (Figure 6a). The field sites studied here are located in the highland region of Santa Cruz Island, which is the central island of the archipelago. Because of its wide elevation gradient (maximum elevation 864 m [42]), it supports vegetation ranging from arid scrub to wet montane forest, with high endemism of woody species in the high-altitude zones [43]. The humid highland region consists of three vegetation types: One dominated by *Scalesia pedunculata*, the second is a mixed forest consisting of *S. pedunculata*, *Psidium galapageium*, *Zanthoxylum fagara*, and *Tournefortia rufo-sericea*, and the third is dominated by *Miconia robinsonia* and ferns [44]. Several factors have altered the highlands of the Galapagos Islands. Vegetation variability associated with El Niño Southern Oscillation influenced the vegetation shift prior to anthropogenic disturbance [45]. During the 1960s, the most transformative anthropogenic disturbance was the introduction of grazing animals, which intensified the agricultural disturbance and shifted the area’s vegetation [44]. Santa Cruz Island has suffered significant human impact, especially in the highland zone, where 76% of vegetation has been transformed by agriculture and plant invasion [46]. Introduced plants on Santa Cruz Island increased between 1987 and 1994 to 76.6%, the highest rate of the inhabited islands of the Archipelago of Galápagos [47]. An inventory reported the three most abundant introduced species were *Psidium guajava* (common guava), *Passiflora edulis* (passion fruit), and *Bryophyllum pinnatum* [48].

Given that the floristic database and history of the location are well documented and that the diversity of plants of this region shows a set of potentially naturally arrived and anthropogenically introduced flora, it is ideal for studying the effects of environmental filtering on plant establishment, potentially increasing our understanding of contemporary plant community assembly, as well as plant invasions.

We analyzed the mycorrhizal status of flora from: (a) The highlands of Santa Cruz Island using the database of the Charles Darwin Research Station (http://checklists.datazone.darwinfoundation.org/vascular-plants/; see also Jaramillo & Guézou, 2011 [49]); and (b) flora of Mache-Chindul mountains of Northwestern Ecuador using the floristic checklist [50]. We determined the plant-mycorrhizal status using Brundrett (2009) [51] and Wang & Qui’s (2006) [52] checklists of the mycorrhizal status of land plants families. Because this is a relatively coarse approach, we selected a subset of plants to experimentally test the mycorrhizal response. 

### 4.2. Experiment 1. Plant Growth Mycorrhizal Response 

We selected representative plant species of the common plant families of the humid highland’s flora of Santa Cruz Island, based upon of seed availability. Twelve native and eight introduced plant species were used in the study (Table S1). As seeds cannot be removed from islands and mycorrhizal inoculum cannot be introduced, we inferred growth response of these plants to mycorrhizal fungi by using whole native soil inoculant combined with fungicide treatment. We grew each species in a randomized blocked design in 3-L pots with either live soil inoculum or sterilized soil. Each species was replicated five times per treatment.

Seeds and soil were collected from three locations (Figure 6b). At each location, we identified two sites dominated by either native or introduced plant species. Descriptions of the geographical locations, vegetation type, and type of disturbance of the sites in study are presented in Table S4, and soil chemical metrics in Table S5, Supporting Information. Soil samples were collected at each of the sites by removing the surface, if present, and collecting the top 12 cm of soil. Soil samples per plot were pooled and handled as one composite sample.

The nonmycorrhizal treatment was treated with benomyl fungicide (20 mg/kg), which was applied at the time of sowing and each month thereafter for the duration of the experiment to suppress arbuscular mycorrhizal colonization [53,54]. 

The pots were grown for four to six months on low benches in a shade-house with light levels similar to those the plants would experience in the highlands. Initial measurements of plant height, stem diameter, and leaf number were recorded. Plants were measured at monthly intervals over the course of the experiment. At harvest, aboveground and belowground material was washed, dried at 40 °C, and weighed to determine biomass. A subsample of roots was taken from each pot to assess the level of AM fungal colonization by clearing them with 10% KOH and staining with trypan blue. Stained roots samples were mounted on slides and root mycorrhizal colonization percentage was assessed microscopically from 120 fields of view per sample at 400× magnification [55]. Specific leaf area (area per unit leaf dry mass) was estimated following Garnier et al. (2001) [56], and leaf area measurements were performed using Image J software (http://rsb.info.nih.gov/ij/). Growth was analyzed using general linear models in SAS. The plant growth mycorrhizal response was estimated as the relative biomass of plant species growing in live soil compared to the fungicide treatment. 

### 4.3. Experiment 2. Plant–Soil Feedback 

To test the strength of plant–soil feedback, we trained and tested the effect of changes in the composition of soil organisms in a two-step experiment [57]. First, we conditioned the soil with five natives and three non-native plant species (Table S1) by growing seedlings with an identical steam-pasteurized 3:1 (v/v) 4-mm sieved lava rock-field soil mixture combined with 10% inoculum from trap cultures. Inoculum from trap cultures was added to increase mycorrhizal spore density, since preliminary observations suggested AM fungi were in low density at our field sites (Table S4). Trap cultures were grown for a year with native (*Sporobulus indicus*) and non-native (*Pennisetum purpureum*) grass species as hosts and field soil from the sites of study, in 20-L pots, in shade-house conditions. Each species was replicated five times per treatment. In the subsequent test experiment, seedlings of each species were grown in pots inoculated with soils from the eight plant species and an uninoculated control in a full factorial design. Seed availability limited the test experiment to five native and one introduced plant species (Table S1). The plants were grown for 10 weeks in shade-house conditions. Each species was replicated 20 times per treatment. The high mortality of one of the native species, *Chiococca alba*, eliminated it from statistical analyses. 

### 4.4. Field Sites and Sampling

Study sites were established at six locations within the geographical range of highlands of Santa Cruz Island. We targeted current locations based on the historical land use and flora information, which represent the main disturbances (naturally colonized or human-disturbed by agricultural practices) and vegetation (dominated by native or introduced vegetation) types in the highlands. The characteristics of the sites are summarized in Table S4. At each site, we conducted vegetation surveys and soil physiochemical analyses, and determined arbuscular mycorrhizal fungi density and diversity. 

The field vegetation survey data was used to confirm the field history of disturbance and flora. We used a Modified-Whittaker nested vegetation sampling method to measure vegetation diversity [58]. We sampled five plots at each study site, representing visually homogeneous vegetation. The plots were 4 × 25 m (100 m^2^), and the distance between plots was generally several hundred meters. In each plot, we established one rectangular 20-m^2^ (10 × 2 m) plot centrally located within the big plot, and five 1-m^2^ (1 × 1) plots randomly assigned and nested within the 100-m^2^ plot (see Figure S1 in Supporting Information). The number of species present in the 100-m^2^ plot with a diameter of > 5 cm at a height of 1 m was recorded. All plant species in the 20-m^2^ plot were recorded. The percentage cover of each herb, shrub, vine, and tree seedling species < 2 m, as well as the density of each, were estimated in the 1-m^2^ plots. Percentage cover was estimated using the following categories: (1) 1–10%, (2) 11–25%, (3) 25–50%, (4) 50–75%, (5) 75–100%.

Shannon’s index (H’), species richness, evenness, and the percentage of native and introduced species were estimated by consolidating the data from all of the subplots. Vegetation origin status was determined using the Charles Darwin Station Flora Database [49]. 

All sampling was conducted by the first author and a local botanist to ensure consistency and accuracy [59]. Sites were sampled between 17 November and 9 December 2010.

Soil samples were collected at each of the vegetation survey plots from five randomly selected subplots and pooled for each plot. Surface litter was removed, if present, and the top 12 cm of soil was sampled to a total bulked volume of 500 cm^3^ per plot. Samples were airdried to constant mass. Soil samples were analyzed for pH, organic matter, and total P. Total C and N were analyzed using a CN analyzer (ECS4010 Nitrogen Analyser, Costech Instruments, Milan, Italy). Soil aggregate stability was analyzed by applying a Water Stable Aggregates (WSA) test [6,60], where each soil sample was separated into six aggregate groups (<0·25 mm, 0.25–0.5 mm, 0.5–1 mm, 1–2 mm, 2–4 mm, and >4 mm) using a rotary sieve for a 2-min cycle. Three technical replicates of approximately 7 g of each aggregate group were separated. Roots, seeds, and rocks were handpicked from each fraction, and the remaining sample was placed on a 250-mm sieve. The soil fractions were hydrated by locating the sieve above a water layer and allowing water to move through the sieve by capillary action for 24 h. After the fractions were moistened on the sieves, they were agitated in distilled water in a sieving apparatus for two cycles of 10 min each at 35 cycles min^−1^. Materials that passed through the sieves were considered to be the water-unstable fraction. Materials that remained on the sieves were soil WSA and sand particles. To separate the WSA from the sand, the sieves were passed through a 1-M NaOH solution at 35 cycles/min^−1^ for two cycles of 10 min each. The sand retained in each sieve was rinsed with distilled water and the WSA–NaOH solution was collected in stainless steel evaporation pans. The pans and the sieves were oven-dried at 110 °C and weighed. The weights were corrected for the weight of the sodium solution fraction that could remain in the samples. The WSA fraction was calculated as the mass of aggregates that remained after wet sieving as a percentage of the initial mass of soil.

The relative density of AM fungi in each soil sample was estimated using a Mycorrhizal Inoculum Potential (MIP) assay. We combined 50 mL of soil inoculum with 150 mL of autoclaved local soil in five replicate conic plant tubes for each site. An additional set of five cones per site received no live inoculum as a control treatment. All cones were seeded with sorghum and watered daily. The cones were grown in a growth chamber at Indiana University (12 h of light and 24 °C). All plants were harvested at 28 days. The roots were washed, and 1 g of them was placed in tissue cassettes to be cleared with 10% KOH and stained with trypan blue. Stained roots samples were mounted on slides and root mycorrhizal colonization percentage was assessed microscopically from 120 fields of view per sample at 400× magnification [55]. The mean of the infection percentage was used as an estimate of the mycorrhizal inoculum potential. 

The AMF diversity was determined by morphological examinations of spores extracted from soil by wet-sieving and decanting following Gerdemann J. W. and Nicholson T. H. (1963) [61]. Spore characterization was performed by mounting them in polyvinyl alcohol-lactic acid-glycerol (PVLG) and a mixture of PVLG-Melzer reagent and examining them with a binocular microscope (1000× magnification). AM fungi were identified to species, whenever possible, based on morphological characters and subcellular structure of spores with the descriptions available at International Culture Collection of (Vesicular) Arbuscular Mycorrhizal Fungi (https://invam.wvu.edu/).

For details of soil chemical analyses and other recorded environmental variables, see Supplementary Methods and Table S5.

### 4.5. Data Analysis and Statistics

To test whether there were significant differences in the mycorrhizal status of the island flora and the site of origin, the database of the mycorrhizal status of floras of both highlands of Santa Cruz Island and Mache-Chindul were analyzed by testing differences in the proportion of species from nonmycorrhizal plant families using general linear models with Poisson error. 

To analyze the factors affecting plant growth response to mycorrhizal fungi colonization, measures of mycorrhizal response of each plant species were calculated as the ratio of shoot biomass when grown in untreated soil over the biomass grown in soil treated with fungicide (Exp. 1) or the ratio of shoot biomass in inoculated soil over that in sterile soil (Exp. 2). We used plant origin (native/naturalized), average growth rate, and plant growth form (woody/non-woody) as predictors of mycorrhizal response in Exp. 1. Exp. 2 was analyzed using general linear models with *a priori* contrasts testing for differences in responsiveness to live soil and strength of feedback [57].

For the field study, we first determined if there were differences in the proportion of soil stable aggregates between the sites dominated by native flora and sites dominated by introduced species. The data was log transformed and then a general linear model analysis was used to examine the effect of site type (anthropogenically disturbed, native, invaded) on WSA, MIP, and AM fungal species richness. A Pearson correlation was used to compare the soil and vegetation variables across sites. Multiple regression was used to determine the best predictors of WSA using the Akaile Information Criterios (AICC) to identify the best model. All statistical tests were done using the program Statistical Analysis System (SAS).

## Supplementary Materials

The following are available online at https://www.mdpi.com/2223-7747/9/1/74/s1, Figure S1. Layout of the plot and subplots of the Modified -Whittaker nested vegetation sampling method used to measure vegetation diversity. Table S1. The species included in the plant growth experiment (1) Plant response and (2) Soil feedback: (a) soil training and (b) feedback test, their scientific name, botanical family, taxon origin and growth habit, Table S2. Analysis of Variance of Feedback Test experiment (Log (1+Aboveground biomass)), Table S3. Mean values of the plant–soil feedback experiment. Values represent the mean ± SE. Table S4. Characteristics of the sites of the study. Geographical location, vegetation type and type of disturbance of the sites in study, Table S5. Soil chemical analysis of the sites of study described in Table S4. Figure S1. Layout of the plot and subplots of the Modified -Whittaker nested vegetation sampling method used to measure vegetation diversity.
