# Supplementary material for: Symbionts as Filters of Plant Colonization of Islands: Tests of Expected Patterns and Environmental Consequences in the Galapagos
Source: Plants (Basel). 2020 Jan 7;9(1):74. doi: 10.3390/plants9010074 (PMC7020428; doi:10.3390/plants9010074)
Supplement: Supplementary file 1 [file plants-09-00074-s001.pdf]

Table 1. The species included in the plant growth experiment 1) Plant response and 2) Soil feedback: a) soil training and b)feedback test, their scientific name, botanical family, taxon origin and growth habit.

**ID**	**Scientific name**	**Botanical Family**	**Taxon origin**	**Taxon sub-origin**	**Growth habit**	**Experiment**
Ca	*Chiococa alba*	Rubiaceae	Native	Indigenous	Shrub	1,2a
CaI	*Coffea arabica*	Rubiaceae	Introduced	Escaped	Shrub	1
Ce	*Cestrum auriculatum*	Solanaceae	Introduced	Escaped	Shrub	1
Co	*Cedrela odorata*		Introduced	Escaped	Tree	2a
Cp	*Cinchona pubescens*	Rubiaceae	Introduced	Escaped	Tree	1
Cs	*Croton scouleri Var. grandifolius*	Euphorbiaceae	Native	Endemic	Tree	1
Mc	*Momordica charantia*	Cucurbitaceae	Introduced	Escaped	Vine	1
Pd	*Pleuropetalum darwinii*	Amaranthaceae	Native	Endemic	Shrub	1
Pe	*Passiflora edulis*	Passifloraceae	Introduced	Escaped	Vine	1
Pg	*Psidium guajava*	Myrtaceae	Introduced	Escaped	Tree	1,2a,b
Pgg	*Psidium galapageium*	Myrtaceae	Native	Endemic	Tree	1,2a,b
Pp	*Pennisetum purpureum*	Poaceae	Introduced	Escaped	Herb	1,2a
Pr	*Psychoria rufipes*	Rubiaceae	Native	Endemic	Shrub	1,2a,b
Ps	*Passiflora suberosa*	Passifloraceae	Native	Indigenous	Vine	1
Si	*Sporobulus indicus*	Poaceae	Native	Indigenous	Vine	1,2a,b
So	*Senna occidentalis*	Caesalpinaceae	Native	Indigenous	Shrub	1
Sp	*Scalesia pedunculata*	Asteraceae	Native	Endemic	Tree	1,2a,b
Spi	*Senna pistaciifolia*	Caesalpinaceae	Native	Indigenous	Shrub	1
Tr	*Tournefortia rufo-sericea*	Boraginaceae	Native	Endemic	Shrub	1
Uu	*Urtica urens*	Urticaceae	Introduced	Questionable I.	Herb	1
Zf	*Zanthoxylum fagara*	Rubiaceae	Native	Indigenous	Shrub	1

Table 2. Analysis of Variance of Feedback Test experiment (Log (1+Aboveground biomass)).

Source	DF	Type III SS	Mean Square	F Value	Pr > F
**L_1*Sp_Id**	5	1.10028461	0.22005692	3.69	0.0033
**Sp_Id**	4	2.23054014	0.55763504	9.35	<.0001
**soiltreat**	8	1.34648911	0.16831114	2.82	0.0056
**live vs sterile**	1	0.02079965	0.02079965	0.35	0.5555
**Sp_Id*soiltreat**	32	4.20686737	0.13146461	2.21	0.0006
**live vs sterile*native**	1	0.31358198	0.31358198	5.26	0.023
**feedback*native**	1	0.35850287	0.35850287	6.01	0.0151
**feedback of sp1**	1	0.50231212	0.50231212	8.43	0.0042
**feedback of sp2**	1	0.01937574	0.01937574	0.33	0.5693
**feedback of sp3**	1	0.10861239	0.10861239	1.82	0.1788
**feedback of sp4**	1	0.05088547	0.05088547	0.85	0.3568
**feedback of sp5**	1	0.02306165	0.02306165	0.39	0.5347
**Error**	182	10.84952725	0.05961279		

Table S3. Mean values of the plant–soil feedback experiment. Values represent the mean ± SE. For the statistical analysis, see S2. Ca *Chiococa alba,* Pg *Psidium guajava,* Pgg *Psidium galapageium,* Pp *Pennisetum purpureum,* Pr *Psychoria rufipes,* Si *Sporobulus indicus,* Sp *Scalesia pedunculata.* Information about the flora botanical family, taxon origin and growth habit is presented in Table 1.

Plant Id	Soil treatment	Labove LSMEAN	Standard Error	Pr > |t|
Pg	Pg	**0.59080366**	0.14758207	<.0001
	Pgg	1.16755638	0.10445184	<.0001
	Pr	1.01286324	0.14068893	<.0001
	Si	0.69780643	0.14068893	<.0001
	Sp	1.43862346	0.10445184	<.0001
	XCa	1.12698647	0.10445184	<.0001
	XCo	1.04747777	0.14068893	<.0001
	XPp	0.85396742	0.19237759	<.0001
	XXC	0.70177873	0.14068893	<.0001
Pgg	Pg	0.40361728	0.18924185	0.0343
	Pgg	0.54511933	0.12033788	<.0001
	Pr	0.6542746	0.18924185	0.0007
	Si	0.68887641	0.16036339	<.0001
	Sp	0.64214158	0.2019411	0.0017
	XCa	0.45252838	0.11177707	<.0001
	XCo	0.53619564	0.18924185	0.0051
	XPp	0.38522047	0.18924185	0.0432
	XXC	0.47404782	0.11177707	<.0001
Pr	Pg	1.40811653	0.22586838	<.0001
	Pgg	0.69943943	0.10592432	<.0001
	Pr	0.96518677	0.12333193	<.0001
	Si	0.98363927	0.13749439	<.0001
	Sp	0.88043427	0.10189043	<.0001
	XCa	1.29127913	0.15920428	<.0001
	XCo	1.28199082	0.15678918	<.0001
	XPp	1.28403247	0.15010941	<.0001
	XXC	1.1512548	0.24282688	<.0001
Si	Pg	1.49033873	0.19651831	<.0001
	Pgg	1.40163355	0.16687478	<.0001
	Pr	1.84852365	0.27993089	<.0001
	Si	1.44431291	0.22017554	<.0001
	Sp	1.82420619	0.25998368	<.0001
	XCa	1.85248341	0.28721893	<.0001
	XCo	1.69024501	0.17513159	<.0001
	XPp	1.54579252	0.18344403	<.0001
	XXC	1.7364868	0.22130609	<.0001
Sp	Pg	1.15942636	0.11078079	<.0001
	Pgg	1.58193245	0.11616811	<.0001
	Pr	1.28300097	0.10010498	<.0001
	Si	1.06983266	0.11416082	<.0001
	Sp	1.48516327	0.10951158	<.0001
	XCa	1.25324828	0.10727506	<.0001
	XCo	1.41608775	0.17505192	<.0001
	XPp	1.63877323	0.10990013	<.0001
	XXC	1.37064061	0.10727506	<.0001

Table S4. Characteristics of the study sites: type of disturbance, vegetation type, introduced and native plant species, plots and their geographical location .

Site	Disturbance Type	Vegetation Type	Introduced Plant species	Native Plant Species	Plot	Latitude	Longitud
CM	Invaded	Introduced	*Psidium guajava*<br>*Tradescantia fluminensis*<br>*Cestrum auriculatum*<br>*Psidium guajava*<br>*Passiflora edulis*	*Croton scouleri*<br>*Psychotria rufipes*<br>*Psidium galapageium*<br>*Zanthoxylum fagara*<br>*Ichnantus nemorosus?*<br>*Thelypteris sp.*<br>*Asplenium auritum*<br>*Tournefortia rufo-sericea*<br>*Passiflora colinvauxii*<br>*Blechum pyramidatum*<br>*Spermacoce remota*<br>*Chiococca alba*<br>*Blechnum occidentale*	1	-0.63755	-90.2997
					2	-0.63735	-90.2993
					3	-0.63737	-90.2989
					4	-0.6372	-90.2987
					5	-0.63755	-90.2987
CMR	Disturbed	Introduced	*Pennisetum purpureum*	N/A	1	-0.6397	-90.2899
					2	-0.63962	-90.29
					3	-0.63952	-90.29
					4	-0.63942	-90.2901
					5	-0.63933	-90.2901
F	Invaded	Introduced	*Cedrela odorata*<br>*Cordia alliodora*<br>*Rubus niveous*<br>*Paspalum conjugatum*<br>*Cestrum auriculatum*	*Drymaria monticola*<br>*Kyllinga brevifolia*<br>*Mecardonia procumbens*<br>*Brachiaria multiculma*	1	-0.65853	-90.4092
					2	-0.6587	-90.4091
					3	-0.65872	-90.4094
					4	-0.65893	-90.4095
					5	-0.65882	-90.4098
FR	Disturbed	Introduced	*Acmella sodiroi*<br>*Desmodium glabrum*<br>*Digitaria ciliaris*<br>*Eleusine indica*<br>*Hyptis pectinata*<br>*Oxalis corniculata*<br>*Paspalum conjugatum*<br>*Pennisetum purpureum*<br>*Plantago major*<br>*Pseudelephantopus spiralis*	*Drymaria monticola*<br>*Kyllinga brevifolia*<br>*Mecardonia procumbens*<br>*Brachiaria multiculma*	1	.	.
					2	.	.
					3	.	.
					4	.	.
					5	.	.

			Solanum americanum<br>*Astrephia chaerophylloides*<br>*Verbena litoralis*				
GA	Native	Native:	*Cestrum auriculatum*<br>*Cinchona pubescens*<br>*Ichnanthus nemorosus*<br>*Rubus niveus*	*Megalastrum pleiosorus*	1	-0.62965	-90.367
				Passiflora colinvauxii<br>*Pilea baurii*	2	-0.62967	-90.3673
				Psychotria rufipes<br>*Sida hederifolia*	3	-0.62973	-90.3679
				Tournefortia rufo-sericea	4	-0.62985	-90.3682

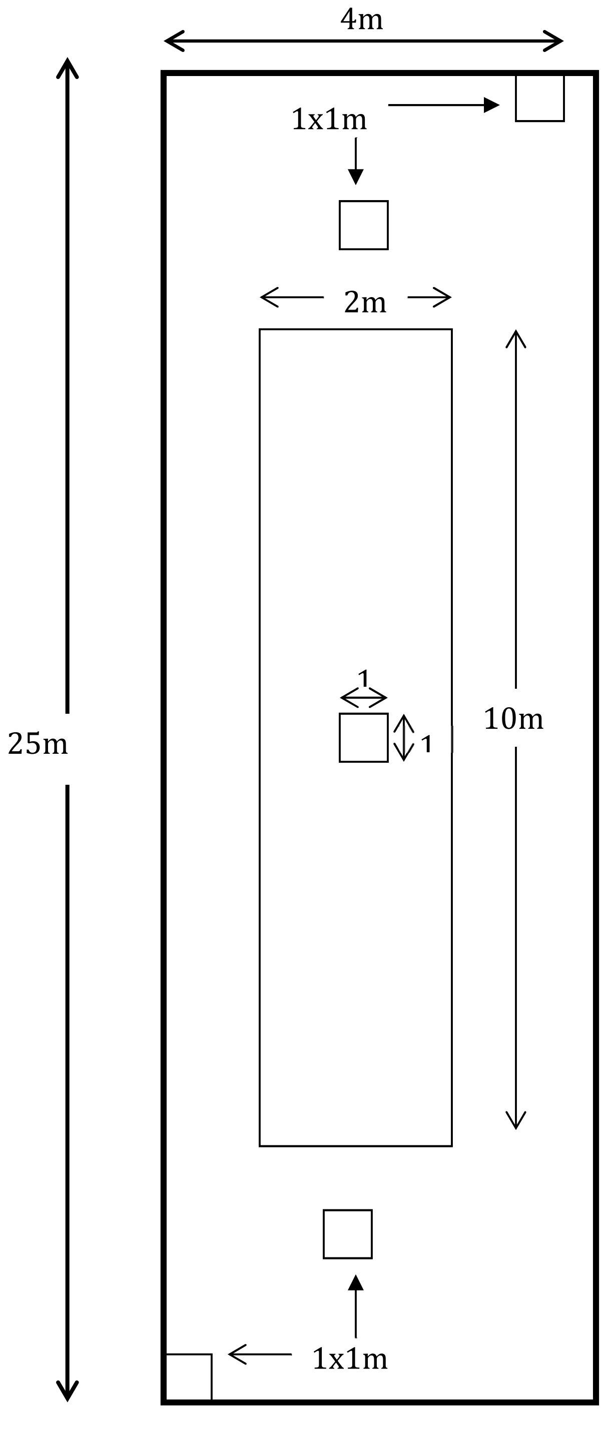

Figure 1. Layout of the plot and subplots of the Modified -Whittaker nested vegetation sampling method used to measure vegetation diversity.
